# Recombinant human plasma phospholipid transfer protein (PLTP) to prevent bacterial growth and to treat sepsis

**DOI:** 10.1038/s41598-017-03285-9

**Published:** 2017-06-08

**Authors:** Valérie Deckert, Stéphanie Lemaire, Pierre-Jean Ripoll, Jean-Paul Pais de Barros, Jérôme Labbé, Caroline Chabert-Le Borgne, Véronique Turquois, Guillaume Maquart, Delphine Larose, Nicolas Desroche, Franck Ménétrier, Naig Le Guern, Lorène J. Lebrun, Catherine Desrumaux, Thomas Gautier, Jacques Grober, Charles Thomas, David Masson, Louis-Marie Houdebine, Laurent Lagrost

**Affiliations:** 1 INSERM LNC, UMR1231 Dijon, France; 2 University Bourgogne Franche-Comté, LNC UMR1231 Dijon, France; 3 LipSTIC LabEx, Fondation de Coopération Scientifique Bourgogne Franche-Comté, Dijon, France; 4 University Hospital of Dijon, Dijon, France; 5 Pharming Group N.V. Branch, Evry, France; 6 Nexidia, Dijon, France; 7 CNRS UMR6265, INRA UMR1324, Centre des Sciences du Goût et de l’Alimentation, F-21000 Dijon, France; 8 AgroSup Dijon, Dijon, France; 9 INSERM U1198, University Montpellier, Montpellier, France; 10 INRA, Development and Reproductive Biology, Jouy en Josas, France

## Abstract

Although plasma phospholipid transfer protein (PLTP) has been mainly studied in the context of atherosclerosis, it shares homology with proteins involved in innate immunity. Here, we produced active recombinant human PLTP (rhPLTP) in the milk of new lines of transgenic rabbits. We successfully used rhPLTP as an exogenous therapeutic protein to treat endotoxemia and sepsis. In mouse models with injections of purified lipopolysaccharides or with polymicrobial infection, we demonstrated that rhPLTP prevented bacterial growth and detoxified LPS. In further support of the antimicrobial effect of PLTP, PLTP-knocked out mice were found to be less able than wild-type mice to fight against sepsis. To our knowledge, the production of rhPLTP to counter infection and to reduce endotoxemia and its harmful consequences is reported here for the first time. This paves the way for a novel strategy to satisfy long-felt, but unmet needs to prevent and treat sepsis.

## Introduction

Plasma phospholipid transfer protein (PLTP) is ubiquitously expressed in vertebrate species. It has the ability to bind and transfer a number of amphipathic compounds, including phospholipids, unesterified cholesterol, diacylglycerides and tocopherols^1^. Phylogenetic analyses revealed that PLTP belongs to the lipid transfer/lipopolysaccharide binding protein (LT/LBP) gene family as do cholesteryl ester transfer protein (CETP), lipopolysaccharide binding protein (LBP) and bactericidal permeability increasing protein (BPI), which are involved in innate immunity^2, 3^. Like most other members of the related palate-lung epithelial clone (PLUNC) superfamily of genes (known to constitute the first barrier of defence against pathogens in the upper airways)^2^, PLTP was recently found to bind and transfer lipopolysaccharides (LPS), which are located in the outer wall of gram negative bacteria^4–8^. Consistently, recent studies have provided data supporting the key role of PLTP in the lipoprotein-mediated reverse LPS transport pathway through which purified LPS aggregates can be disrupted, transferred to lipoprotein vehicles and transported to the liver for elimination in the bile^6, 7^. Earlier *in vitro* and *in vivo* studies revealed that the PLTP-mediated transfer of LPS to lipoproteins results in the neutralization of the proinflammatory properties of LPS and in its elimination from the body. In further support of the pathophysiological relevance of the PLTP-mediated detoxification of LPS, PLTP-knocked out mice were less able than wild-type mice with naturally elevated PLTP expression to get rid of purified LPS^6^. This suggests that recombinant PLTP might constitute a novel and relevant therapeutic tool for the prevention and treatment of LPS-mediated inflammation.

Although earlier studies described the production of human recombinant PLTP (rhPLTP) in eukaryotic cells^9–11^, only low levels of active rhPLTP could be produced in bioreactors, thus hampering the possibility of using it at therapeutic levels in sepsis. Importantly, PLTP is a complex glycosylated protein, with six distinct N-glycosylation sites which are essential for normal activity^12, 13^. In earlier studies, transgenic rabbits successfully produced recombinant human proteins^14–17^, some of which are currently under investigation in human therapeutics (alpha-glucosidase^18^, C1-esterase inhibitor^19^). The production of recombinant proteins by rabbits offers a number of advantages: easy generation of transgenic founders and offspring, high fertility, post-translational modifications close to what occurs in humans, insensitivity to prion diseases, and a low risk of transmission of severe diseases to humans. Mammary glands are the organs of choice to express valuable recombinant proteins in the transgenic rabbit bioreactor. Rabbit milk is easily collected in large volumes, it is naturally extremely rich in proteins (100 to 140 g/l), and each lactating female rabbit can produce up to 200 g of milk per day^20^. The expression of human recombinant proteins in rabbit milk can be achieved successfully with promoters from milk protein genes, including the whey acidic protein (WAP) promoter. The optimization of transgene constructs under the control of the WAP promoter could be obtained by including enhancers, insulators, introns and transcription terminators to allow protein expression in a reliable manner^21^. Given the lipophilic nature of PLTP and its affinity for liposome and lipoprotein structures, milk could constitute a favorable environment for the preservation of its stability and activity. There is, nonetheless, a potential drawback due to putative sequestration of the recombinant protein in milk fat globules. At this stage, it is unknown whether the production of recombinant human PLTP (rhPLTP) in transgenic rabbit milk is a safe and promising strategy to generate large amounts of PLTP for therapeutic purposes.

In the present study, active rhPLTP was produced in the milk of new lines of transgenic rabbits with the human PLTP sequence placed under the control of the WAP promoter. The resulting purified rhPLTP was found to display the main features of the native protein. Here, it was successfully used as an exogenous therapeutic protein to prevent bacterial growth and to detoxify LPS *in vivo*. Consistent beneficial properties of rhPLTP were obtained in mouse models with purified LPS injection or with polymicrobial infection. Finally, and in further support of the antimicrobial effect of PLTP, PLTP-knocked out mice were found to be less able than wild-type (WT) mice to fight against sepsis.

## Results

### Production and characterization of human recombinant PLTP

**Figure 1:**
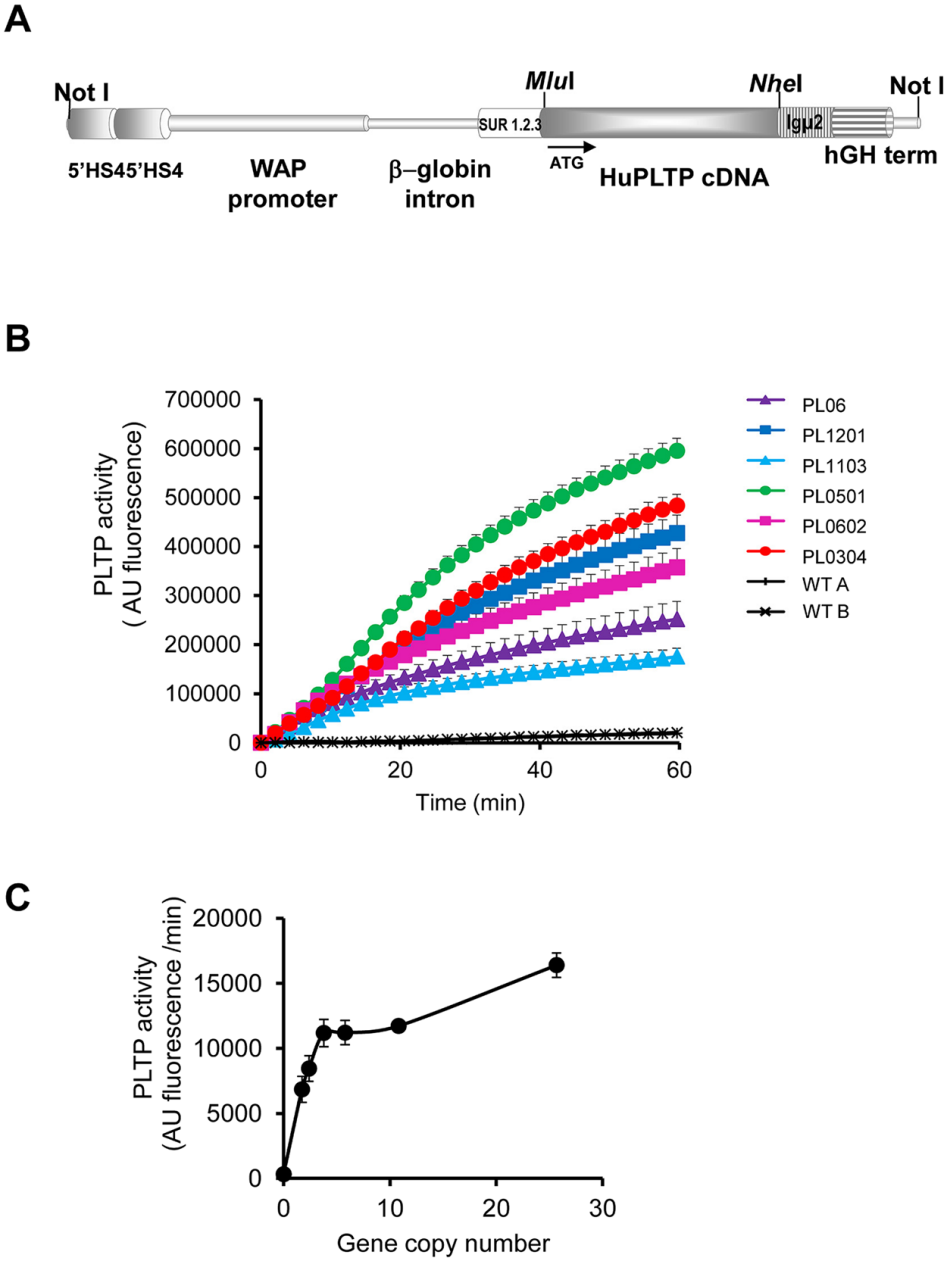
Generation of HuPLTPTg rabbit lines. (**A**) Structure of the human PLTP gene construct. The vector contains a dimer of the 5′HS4 insulator from the chicken β-globin locus; the 6.3 kbp rabbit WAP promoter; the second intron of the rabbit β-globin gene containing an enhancer; a second enhancer (SUR 1.2.3) containing the 5′UTR sequence of the SV40 early gene fused with the R region and the beginning of the U5 region of HTLV-1; the insertion site (with *Mlu*I and *Nhe*I restriction sites) of the human PLTP cDNA; a third transcription enhancer (Igμ2), derived from mu region of the mouse IgG heavy chain and finally the human growth hormone transcription terminator. (**B**) Human PLTP transgene expression in the rabbit mammary gland produced phospholipid transfer activity in the milk. Phospholipid transfer activity was measured by fluorimetric assay in the milk of wild-type (WT) and transgenic rabbit (PL) lines. (**C**) Phospholipid transfer activity in PLTPTg rabbit milk was related to the number of PLTP gene copies. PLTP activity was assayed in milk collected from six different PLTPTg rabbit lines. Data are means ± sem (n = 4–5 days of lactation).

We generated a new model of transgenic rabbit in which an optimized sequence corresponding to human PLTP cDNA was placed under the control of the rabbit whey acidic protein (WAP) promoter (HuPLTPTg rabbits) (Fig. 1A). In this model, human PLTP is produced in the mammary gland and is secreted in the milk of rabbit does. Human PLTP transgene expression in rabbit mammary glands resulted in the production of transgenic milk with elevated levels of PLTP activity (Fig. 1B). The level of rhPLTP production in milk depends on the number of integrated transgene copies (Fig. 1C). The relationship between PLTP activity and the number of PLTP gene copies is linear up to 5–7 copies, and it becomes asymptotic beyond 7 copies, reaching a maximal production rate. This is probably due to titration of the transcription factors which are present in insufficient amounts to stimulate a high number of transgene copies (Fig. 1C).

**Figure 2:**
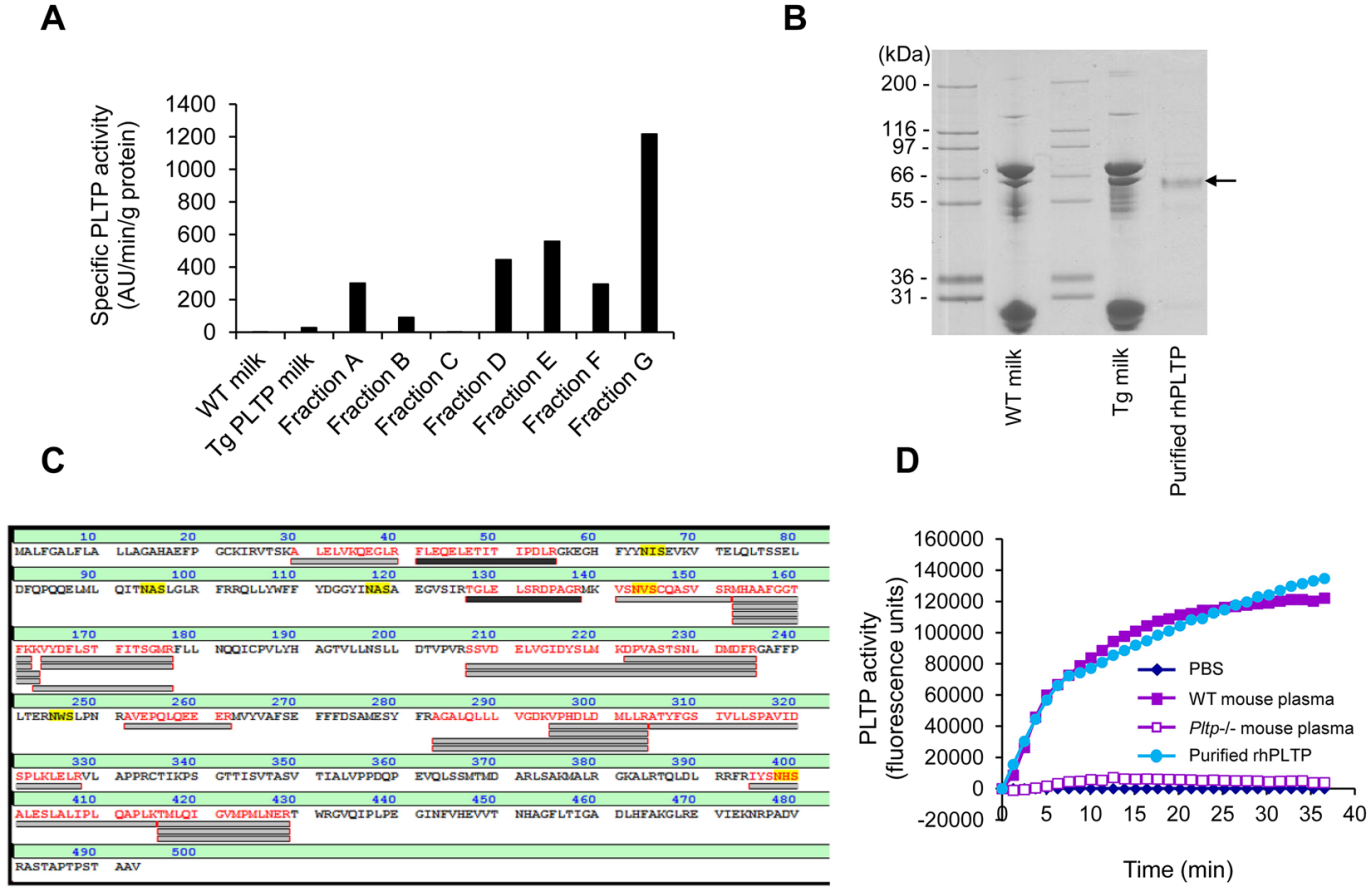
Purification and characterization of recombinant human PLTP from PLTPTg rabbit milk. (**A**) After FPLC elution of clarified milk proteins on a Heparin Sepharose 6 Fast Flow column, using a discontinuous NaCl gradient with 100 mM step increases, different fractions (A–G) were collected at different NaCl concentrations and analyzed for specific PLTP activity. The fraction exhibiting the highest specific PLTP activity (G) was eluted at the 300 mM NaCl step. (**B** and **C**) Electrophoretic analysis of purified rhPLTP (**B**) shows a sharp, 60 kDa band which was analyzed by MALDI-TOF/TOF MS and a database search. The protein (arrow) was identified as human PLTP according to the matched peptides which are shown in bold red (**C**). (MW: 54.7 kDa. Number of matching peptides: 24. Sequence coverage: 39.8%. Score: 182 for a significant matching score above 50). (**D**) Comparison of the phospholipid transfer activities of active purified rhPLTP, wild-type (WT) mouse plasma, *Pltp*−/− mouse plasma, and PBS buffer. The active rhPLTP shows phospholipid transfer activity of the same magnitude as that in WT mouse plasma.

After the precipitation of caseins, centrifugation and filtration, clarified milk obtained from transgenic rabbits was subjected to chromatography affinity on a Heparin Sepharose column using a discontinuous NaCl gradient. Several eluted fractions corresponding to successive 280 nm absorbance peaks were collected and tested for specific PLTP activity (fractions A to G in Fig. 2A). Among these fractions, fraction G eluted at 300 mM NaCl displayed the highest specific PLTP activity, which was 44 times higher than that in raw transgenic rabbit milk. Purified rhPLTP was found to match the human PLTP sequence according to MALDI-TOF/TOF MS analysis (Fig. 2B,C), with 24 distinct matching peptides, a sequence coverage of 39.8%, a calculated MW of 54.7 kDa, and a significant matching score of 182. These data indicate that the production and purification processes set up in the present study led to the production of active human recombinant PLTP (rhPLTP). Importantly, and as shown in Fig. 2D, time course curves of phospholipid transfers with purified rhPLTP fractions using synthetic liposome donors were found to be similar to those obtained with the plasma of WT mice, which naturally expresses the highest level of plasma PLTP activity among mammal species studied so far^22^. Milk from the highest transgenic expressors of PLTP contained up to 1 g/l of PLTP, which is 250 times greater than known PLTP concentrations in healthy human plasma^23^.

### Prevention of endotoxic shock by the injection of exogenous rhPLTP into mice

Previous data from our laboratory have shown that disabling the endogenous gene for PLTP in mice (*Pltp*−/−) leads to a reduction in their ability to neutralize and detoxify purified LPS compared with WT mice. After the injection of a lethal dose of purified LPS, death was found to occur earlier in *Pltp*−/− than in WT mice^6^.

**Figure 3:**
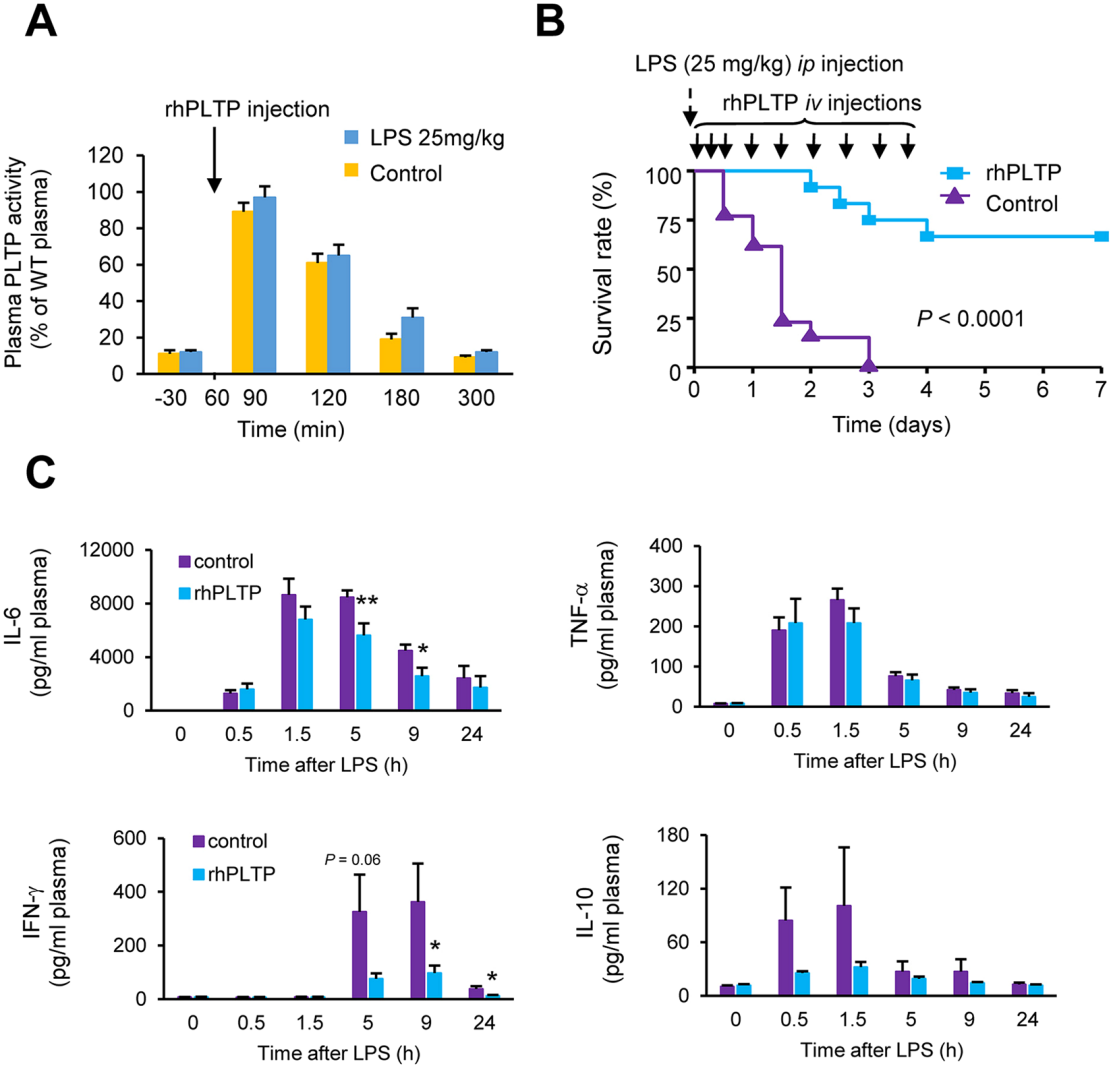
rhPLTP protected *Pltp*−/− mice against inflammation and mortality induced by purified LPS injection. (**A**) Time course of enhanced PLTP activity in the plasma of *Pltp*−/− mice after a single *iv* injection of rhPLTP. *Pltp*−/− mice were *ip* injected with saline or LPS (25 mg/kg) and 1 hour later they received an *iv* injection of rhPLTP (25 µg in a volume of 200 µl). PLTP activity was measured in plasma before and at the indicated time after LPS or saline injection and expressed as a percentage of WT mice plasma activity used as a reference. Data are means ± sem (n = 5 mice per group). (**B**) Repeated *iv* injections of rhPLTP increased survival following endotoxic shock. *Pltp*−/− mice received several *iv* injections of vehicle (control) or rhPLTP (25 µg in a volume of 200 µl) 30 min, 5 h, 10 h, 24 h, 32 h, 48 h, 56 h, 72 h, and 80 h after receiving a single dose of LPS (25 mg/kg of body weight, intraperitoneally). Survival rates were analyzed by the Kaplan-Meier method and compared using log rank test (n = 12–13 mice per group). (**C**) Repeated *iv* injections of rhPLTP prevented the production of inflammatory cytokines after LPS-induced endotoxemia. *Pltp*−/− mice were *ip* injected with a sublethal, 5 mg/kg LPS dose. Subsequently, they received several *iv* injections of vehicle (control) or rhPLTP (25 µg in a volume of 200 µl) at 10 min, 2 h, 4 h, 6 h and 8 h time points. Plasma samples, harvested from *Pltp*−/− mice before LPS and 30 min, 1.5 h, 5 h, 9 h and 24 h after LPS administration were assayed using a Milliplex mouse cytokine panel (IL-6, TNF-α, IFN-γ and IL-10) (n = 8 mice per group, Mann-Whitney test). Data are means ± sem. *P < 0.05, **P < 0.01.

In a first attempt to determine whether an exogenous supply of rhPLTP could facilitate LPS detoxification and increase resistance to endotoxic shock, *Pltp*−/− mice were injected intraperitoneally (*ip*) with purified LPS from *E*. *coli* O55:B5 or saline, followed by a single intravenous injection of rhPLTP containing 25 µg of active PLTP in a volume of 200 µl. Thirty minutes after the rhPLTP injection, plasma PLTP activity was drastically increased (by 8 fold) in both groups; it then decreased progressively to return to the baseline level 4 hours later (Fig. 3A). These results show that the injection of exogenous PLTP into mice led to a substantial and transient rise in plasma PLTP activity, which persisted for a few hours. It is noteworthy that the administration of rhPLTP to *Pltp*−/− mice increased the level of plasma PLTP activity to the level routinely found in WT mice.

In a second set of experiments, *Pltp*−/− mice received a lethal injection of purified LPS (25 mg/kg) via the intraperitoneal route at time 0 followed by repeated intravenous injections of PBS buffer or rhPLTP (25 µg of active PLTP in a volume of 200 µl) over 4 days. Mortality rates were recorded over a period of 7 days after LPS injection. As shown in Fig. 3B, successive injections of rhPLTP during the first few hours following the LPS injection considerably extended survival in these mice. Whereas all of the *Pltp*−/− mice injected with PBS died within 3 days after LPS injection, 65% of *Pltp*−/− mice treated with rhPLTP were still alive after 7 days. Consistent with their increased survival, *Pltp*−/− mice treated with successive injections of rhPLTP showed a weaker inflammatory response as assessed by lower levels of IL-6 and IFN-γ (Fig. 3C).

### Prevention of sepsis by injecting exogenous rhPLTP into *Pltp*−/− mice with polymicrobial infection

**Figure 4:**
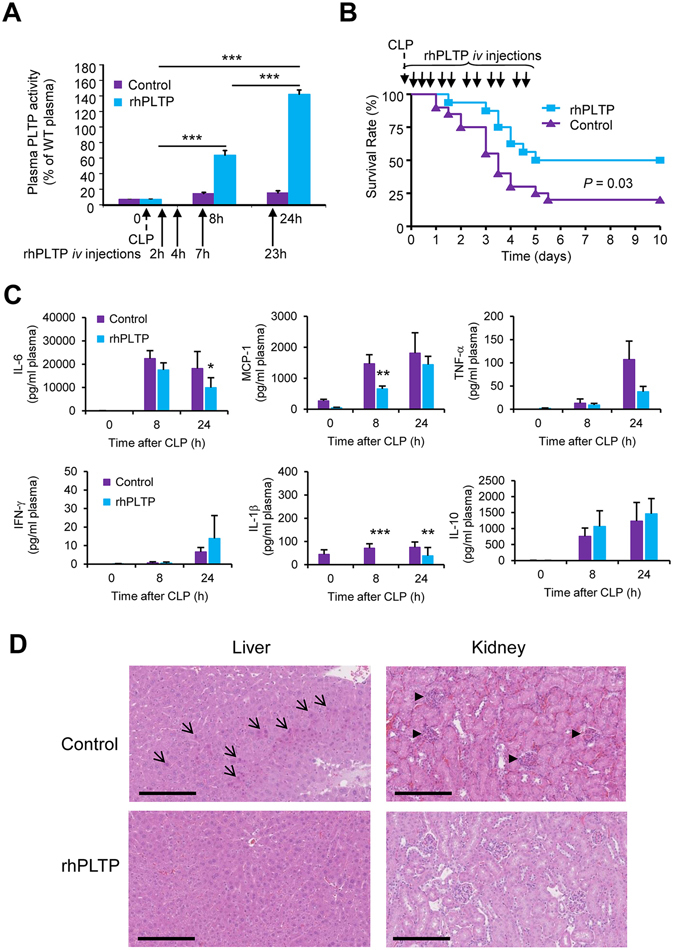
rhPLTP protected against inflammation and death in *Pltp*−/− mice with polymicrobial sepsis. (**A**) Repeated *iv* injections of rhPLTP after CLP produced a cumulative effect on plasma PLTP activity. *Pltp*−/− mice received *iv* injections of vehicle (control) or rhPLTP (25 µg in a volume of 200 µl) 2 h, 4 h, 7 h and 23 h after CLP. PLTP activity was measured in plasma before and 8 h and 24 h after CLP and expressed as a percentage of WT mouse plasma activity used as a reference (n = 8 mice per group, ANOVA with Tukey’s multiple comparisons test). (**B**) Repeated *iv* injections of rhPLTP increased survival after CLP. *Pltp*−/− mice received several intravenous injections of rhPLTP (25 µg in a volume of 200 µl) or vehicle at 2 h, 4 h, 7 h, 23 h, 32 h, 48 h, 56 h, 72 h, 80 h, 96 h, 104 h after CLP. Survival rates were analyzed by the Kaplan-Meier method and compared using the log rank test (n = 16–20 mice per group). (**C**) Repeated *iv* injections of rhPLTP prevented the production of inflammatory cytokines (IL-6, MCP-1, TNF-α, IL-1β) after CLP. *Pltp*−/− mice received several *iv* injections of vehicle (control) or rhPLTP (25 µg in a volume of 200 µl) 2 h, 4 h, 7 h, and 23 h after CLP. Plasma samples, harvested from *Pltp*−/− mice before CLP, 8 h and 24 h after CLP, were assayed using a Milliplex mouse cytokine panel (n = 15–19 mice per group, Mann-Whitney test). Data are means ± sem. *P < 0.05, **P *<* 0.01, ***P < 0.001. (**D**) Histological examination of liver and kidney of *Pltp*−/− mice 24 h after CLP showed a protective effect of rhPLTP injections. The organs were collected 24 h after CLP, and fixed with paraformaldehyde prior to HE staining. The arrows indicate the presence of hepatocyte clusters with acidification. The arrowheads indicate damaged glomeruli. Representative photographs of 5 mice per group are shown (original magnification x400). Scale bars are equivalent to 200 µm.

Because the strategy of repeated rhPLTP injections proved to be successful in down-regulating the inflammatory response and its harmful consequences in the purified LPS injection model, we turned towards a polymicrobial infection model as induced by cecal ligation and puncture (CLP), which is recognized as a clinically relevant animal model of sepsis^24^. In *Pltp*−/− mice, we observed that repeated intravenous (*iv*) injections of rhPLTP after CLP resulted in incremental increases in plasma PLTP activity, even exceeding by 40% the activity measured in the plasma of WT mice 24 hours after CLP (Fig. 4A). Interestingly, the repeated *iv* injections of rhPLTP in *Pltp*−/− mice also had a significant impact (*P* = 0.016) on the PLTP activity of peritoneal fluid which reached 32 ± 5% of that detected in homologous mice plasma (16371 ± 5032 AU in peritoneal fluid *vs* 51263 ± 13165 AU in plasma, n = 4 animals). The rhPLTP administration sequence set up in the present study substantially reduced mortality in *Pltp*−/− mice following CLP (Fig. 4B). Ten days after CLP, 50% of the rhPLTP-treated mice were still alive against only 20% of mice receiving the vehicle. The rhPLTP treatment of *Pltp*−/− mice was associated with a significant reduction in the circulating concentrations of IL-6, MCP-1, and IL-1β as well as a downward trend for TNF-α as observed at 24 h after CLP (Fig. 4C). Liver histology performed 24 hours after CLP on *Pltp*−/− mice (Fig. 4D, left panels) showed more damage in livers from vehicle-injected animals, especially with substantial acidification of hepatocytes undergoing cell death, than in the livers of mice injected with rhPLTP. Kidney histology (Fig. 4D, right panel) revealed a higher amount of injured glomeruli with congestion, hyper-cellularity, compression of capillary loops and reduction of Bowman’s space in vehicle-injected animals than in rhPLTP injected mice.

Overall, repeated injections of rhPLTP during the first days following CLP reduced the production of inflammatory cytokines and organ damage, and extended survival.

### rhPLTP has direct anti-bacterial properties

**Figure 5:**
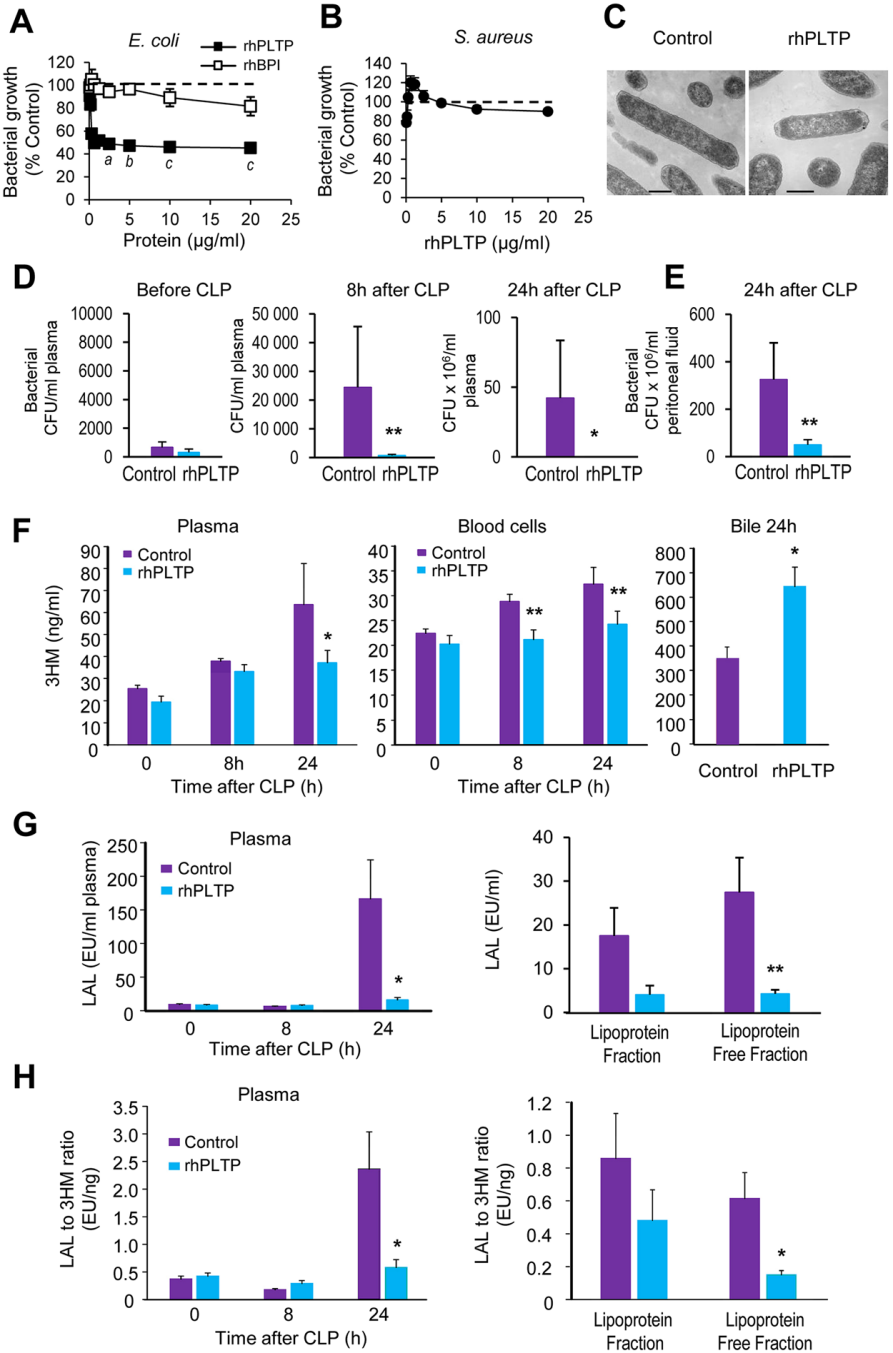
rhPLTP injection reduced the bacterial burden and increased the clearance of LPS from the bloodstream of *Pltp*−/− mice with polymicrobial sepsis. rhPLTP inhibited *E*. *coli* growth *in vitro* to a greater extent than did rhBPI (**A**) but had no effect on *S*. *aureus* (**B**). The effect of rhPLTP and rhBPI on bacterial growth was examined using broth microdilution assay in nutrient MHB. Bacterial growth was determined after 6 h of incubation and expressed as a percentage of control growth (dashed line) *i*.*e*. without rhPLTP and rhBPI (*E*. *coli*: n = 5 independent experiments; *S*. *aureus*: n = 4 independent experiments, Kruskal-Wallis test with Dunn’s multiple comparisons test). ^a^ *P* < 0.05, ^b^ *P* < 0.01, ^c^ *P* < 0.001 significantly different from control growth. (**C**) Representative TEM images of *E*. *coli* after 6 h incubation in MHB in the absence (Control) or in the presence of rhPLTP. Normal ATCC25922 *E*. *coli* (Fig. 5C-Control) display typical Gram-negative structures with intact membranes and high-density cytoplasm. *E*. *coli* exposed to rhPLTP (Fig. 5C-rhPLTP) for 6 h displayed condensation of the cytoplasmic contents. Scale bars, 500 nm. (**D** and **E**) Intravenous rhPLTP administration reduced bacterial burden in blood samples and bacterial load in peritoneal lavages of *Pltp*−/− mice with polymicrobial sepsis. Colony-forming units (CFU) were determined in blood samples (**D**) before CLP, 8 h and 24 h following CLP and in peritoneal lavage fluids (**E**) 24 h after CLP (n = 15 mice per group, Mann-Whitney test). (**F**–**H**) Intravenous rhPLTP administration reduced LPS levels in plasma and blood cells by increasing its biliary excretion in *Pltp*−/− mice after CLP. LPS concentrations (**F**) were determined by direct quantitation of 3-hydroxymyristate (3HM) over a 24 h period following CLP in plasma, blood cells and bile from *Pltp*−/− mice (n = 16–18 mice per group, Mann-Whitney test). LPS biological activity (**G**) in plasma and fractions was quantified by LAL assay (n = 16–18 mice per group, Mann-Whitney test (plasma) and *t* test (fractions)), and LPS activity index (**H**) was calculated as the LAL to 3HM ratio (n = 16–18 mice per group, Mann-Whitney test). Data are means ± sem. *P < 0.05, **P *<* 0.01.

Beyond detoxification properties of LPS, we investigated the anti-bacterial effect of rhPLTP in comparison with rhBPI, *i*.*e*. another LT/LBP member known to reduce bacterial growth. To this end, *E*. *coli* growth was measured in the presence of either rhPLTP or rhBPI using the broth microdilution assay (Fig. 5A). rhPLTP inhibited bacterial growth to a greater extent than did rhBPI. After 6 h of incubation, bacterial growth was reduced by 65 ± 1% in the presence of rhPLTP as compared to only 18 ± 8% in the presence of rhBPI. Under the same experimental conditions, the growth of *S*. *aureus* was not inhibited by rhPLTP (Fig. 5B).

As compared to untreated *E*. *coli*, which displayed a typical Gram-negative structure with an intact membrane and high-density cytoplasm (Fig. 5C – control), rhPLTP-treated cells displayed condensation of the cytoplasmic content and changes in cell membrane morphology, as well as plasmolysis, which is known to involve the outflow of intracellular constituents (Fig. 5C – rhPLTP).

Concerning the bacterial burden in the blood and peritoneal lavage fluid of mice subjected to CLP, recurrent administration of rhPLTP in mice diminished bacterial concentrations in the blood to almost undetectable levels at 8 h and 24 h after CLP (Fig. 5D). After 24 hours, bacterial concentrations were markedly lower in peritoneal lavage fluid of rhPLTP-treated mice than in peritoneal lavage fluid of vehicle-treated mice (Fig. 5E). Since blood cultures obtained after CLP are known to result mainly from the translocation of gram negative bacteria^25^, the direct quantitation of 3-hydroxymyristate (3HM) (*i*.*e*. the most abundant hydroxylated fatty acid of the lipid A moiety of most LPS molecules) was performed by LC/MS/MS^26^. Injecting *Pltp*−/− mice with rhPLTP enhanced LPS clearance after CLP. This was highlighted by the lower 3HM levels detected in the bloodstream of *Pltp*−/− mice treated with rhPLTP *versus* vehicle. A substantial decrease in 3HM was first observed in the blood cells at 8 h and in the plasma at 24 h after CLP (Fig. 5F). Biliary excretion of 3HM was significantly increased (P = 0.04) as illustrated by the nearly two-fold increase in the amounts of 3HM detected in the bile of mice injected with rhPLTP as compared to vehicle (Fig. 5F). The greater efficiency of rhPLTP in LPS detoxification was further supported by the marked inhibition of the biological activity of LPS in the plasma of mice treated with rhPLTP. This was illustrated by the tremendous decreases in LAL activity 24 h post-CLP, in both total and lipoprotein-free plasma (Fig. 5G). In rhPLTP-treated mice, the LPS activity index, calculated as the LAL to 3HM ratio, was decreased in total and lipoprotein-free plasma fractions (Fig. 5H).

### Exogenous rhPLTP maintains its therapeutic properties when injected into WT mice with polymicrobial infection

**Figure 6:**
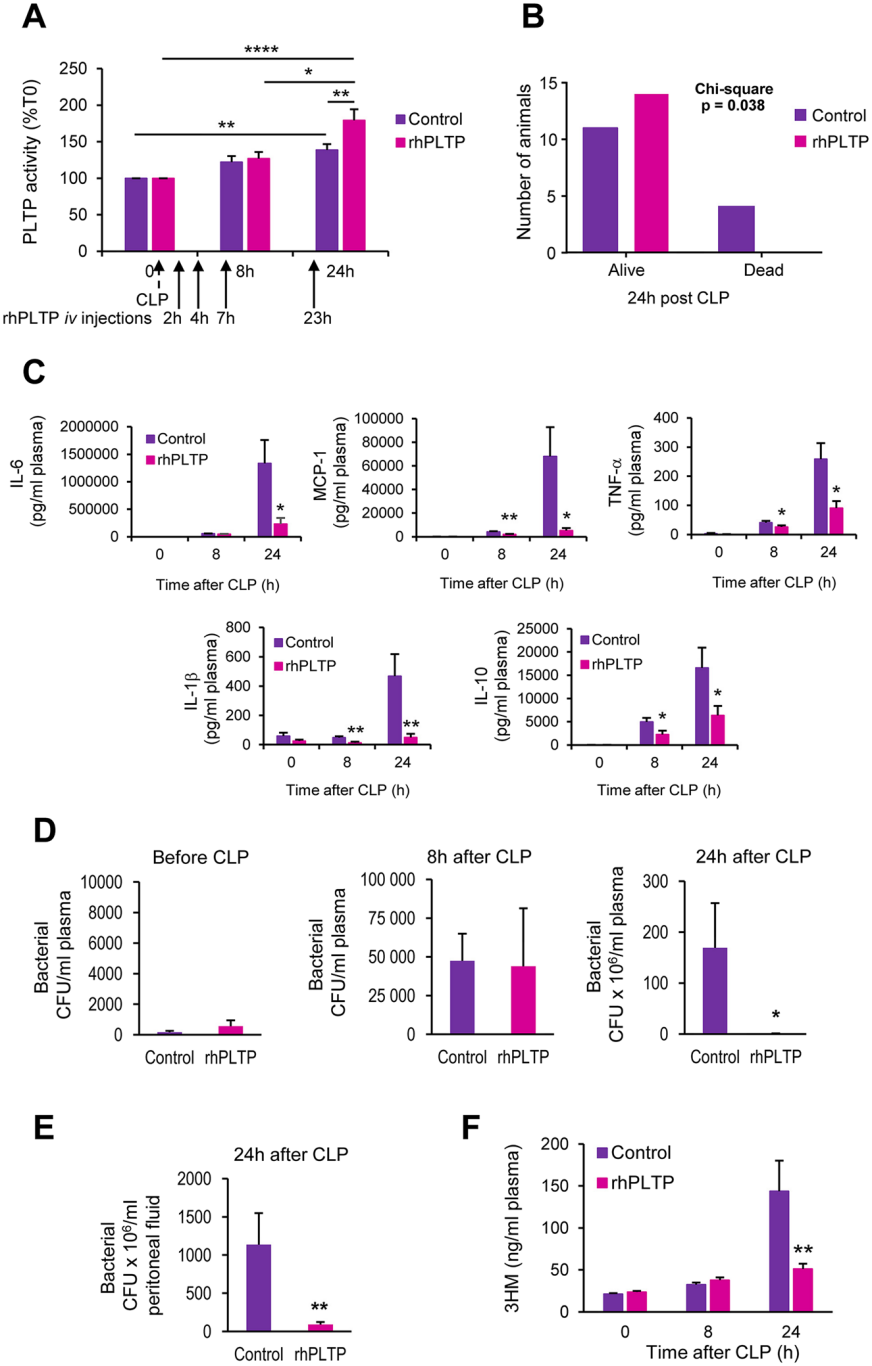
rhPLTP retained its protective effects in WT mice with polymicrobial sepsis. WT mice received *iv* injections of vehicle (control) or rhPLTP (25 µg in a volume of 200 µl) 2 h, 4 h, 7 h and 23 h after CLP. (**A**) Repeated *iv* injections of rhPLTP after CLP induced a significant increase in plasma PLTP activity 24 h after CLP. PLTP activity was measured in plasma before, 8 h and 24 h after CLP and expressed as a percentage of T0 mouse plasma activity (n = 12–14 mice per group, Kruskal-Wallis test with Dunn’s multiple comparisons test). (**B**) Repeated *iv* injections of rhPLTP increased mouse survival after CLP. Numbers of alive and dead animals were compared using the χ^*2*^ test (n = 14–15 mice per group). (**C**) Repeated *iv* injections of rhPLTP prevented the production of cytokines (IL-6, MCP-1, TNF-α, IL-1β, IL-10) after CLP. Plasma samples, harvested from WT mice before CLP, 8 h and 24 h after CLP, were assayed using a Milliplex mouse cytokine panel (n = 12–14 mice per group, Mann-Whitney test). (**D** and **E**) Intravenous rhPLTP administration reduced bacterial burdens in blood and peritoneal lavages of WT mice with polymicrobial sepsis. Colony-forming units (CFU) were determined in blood samples (**D**) before CLP, 8 h and 24 h following CLP and in peritoneal lavage fluids (**E**) 24 h after CLP (n = 12–14 mice per group, Mann-Whitney test). (**F**) Intravenous rhPLTP administration significantly reduced LPS levels in WT plasma after CLP. LPS concentrations were determined by direct quantitation of 3-hydroxymyristate (3HM) over a 24 h period following CLP in plasma from WT mice (n = 12–14 mice per group, Mann-Whitney test). Data are means ± sem. *P < 0.05, **P *<* 0.01.

In WT mice, and in agreement with observations in *Pltp*−/− mice (see above), repeated injections of rhPLTP after CLP resulted in increased plasma PLTP activity (visible after 24 hours – Fig. 6A), reduced mortality 24 hours after CLP (Fig. 6B) and a marked reduction in the circulating concentrations of IL-6, MCP-1, and IL-1β, and in addition here with reductions in TNF-α and IL-10 (Fig. 6C). Again, recurrent administration of rhPLTP in mice diminished bacterial concentrations in the blood and peritoneal lavage 24 h post CLP to levels 250 times lower than those in mice injected with vehicle (Fig. 6D,E). Accordingly, the plasma 3HM level detected in the bloodstream of WT mice treated with rhPLTP was significantly lower (P = 0.006) at 24 h after CLP (Fig. 6F).

### PLTP-knocked out mice are less protected than wild-type mice against polymicrobial sepsis

**Figure 7:**
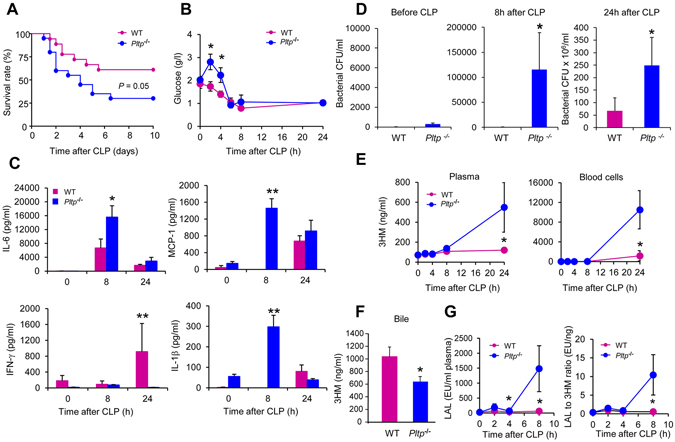
Endogenous PLTP expression protected mice against inflammation and death associated with polymicrobial sepsis. (**A**) PLTP deficiency increased mortality. Age-matched WT and *Pltp*−/− male mice underwent CLP. Data were analyzed using the Kaplan-Meier method, with statistical significance determined using the log rank test (n = 18–20 mice per group). (**B**) PLTP deficiency induced hyperglycaemia post-CLP. Blood glucose concentration was determined with a glucose meter before and at 2 h, 4 h, 6 h, 8 h and 24 h following CLP (n = 5 mice per group, *t* test). (**C**) PLTP deficiency increased the production of the inflammatory cytokines IL-6, MCP-1, and IL-1β and decreased that of IFN-γ. Plasma samples, harvested from WT and *Pltp*−/− mice before CLP, and 8 h and 24 h after CLP, were assayed using a Milliplex mouse cytokine panel (n = 5 mice per group, Mann Whitney test). (**D**) PLTP expression prevented sepsis by decreasing bacterial burden. Colony-forming units (CFU) were determined in blood samples harvested from WT and *Pltp*−/− mice before CLP, 8 h and 24 h following CLP (n = 11–13 mice per group, Mann Whitney test). (**E**-**G**) PLTP expression protected mice against polymicrobial sepsis by limiting the concentration and biological activity of LPS in the bloodstream, by increasing its biliary excretion, resulting in a higher plasma LPS neutralizing capacity. LPS concentrations in plasma and blood cells (**E**) and in bile (**F**) harvested from WT and *Pltp*−/− mice were determined following CLP by direct quantitation of 3HM (plasma and blood cells n = 5–6 mice per group, Mann Whitney test; bile n = 12 mice per group, *t* test). The biological activity of LPS in plasma (**G**) was quantified by LAL assay and the LPS activity index was calculated as the LAL to 3HM ratio (n = 6–7 mice per group, Mann Whitney test). Data are means ± sem. *P < 0.05, **P < 0.01.

In support of the cause-effect relationship between PLTP expression and antimicrobial defense, the resistance of *Pltp*−/− mice to the harmful consequences of sepsis induced by polymicrobial infection was compared with that in WT mice. Whereas 61% of WT mice survived after CLP, 70% of *Pltp*−/− mice died (Fig. 7A). Consistent with the increased mortality of *Pltp*−/− mice, glycaemia in these mice was higher than that in WT mice at 2 h and 4 h after CLP (Fig. 7B). Plasma concentrations of IL-6, MCP-1 and IL-1β increased after CLP with maximal values after 8 h in both WT and *Pltp*−/− mice. In addition, IL-6, MCP-1 and IL-1β peaks were markedly higher in *Pltp*−/− mice than in WT controls (Fig. 7C).

Given the bacteriostatic properties of rhPLTP documented above, we also investigated whether endogenous PLTP expression affected the bacterial burden in the bloodstream. The bacterial concentration in the blood of *Pltp*−/− mice was substantially higher than that in WT mice at 8 h and 24 h following CLP (Fig. 7D). This result is supported by quantification of the LPS concentration. Indeed, the amounts of LPS detected in the plasma and blood cells of *Pltp*−/− mice 24 h after CLP were higher than those in WT counterparts (Fig. 7E). Bile levels of 3HM measured 24 h after CLP were much lower in *Pltp*−/− than in WT mice, indicating a weaker LPS excretion capacity in mice lacking PLTP (Fig. 7F). In accordance with this weaker detoxification efficiency, the biological activity of LPS as quantified by the LAL assay 8 h post CLP was found to be significantly higher (P = 0.04) in the plasma of *Pltp*−/− mice than that in the plasma of WT mice (Fig. 7G). Consistent with the above, the LPS activity index calculated as the LAL to 3HM ratio^26^ was significantly higher (P = 0.04) in *Pltp*−/− than in WT mice.

## Discussion

The present study conducted with genetically engineered mouse and rabbit models showed a dual beneficial effect of plasma PLTP in anti-microbial defenses through its ability to decrease bacterial growth and to accelerate LPS detoxification. The anti-microbial properties could be highly conserved in a preparation of recombinant human PLTP, which was obtained from the mammary gland of a new line of PLTP transgenic rabbits, which were used as a bioreactor. Large scale production of active rhPLTP paves the way for a novel therapeutic strategy in the prevention and treatment of sepsis.

In an attempt to satisfy the long-felt, but unmet needs to prevent and treat sepsis, the present study aimed to demonstrate that recombinant human PLTP could be a potential therapeutic agent. To achieve this goal, active rhPLTP needs to be produced in sufficient amounts for effective use. The rabbit is a highly relevant and our preferred bioreactor because our laboratory has expertise in rabbit genetic engineering^27^, the model depends on a readily available, relevant and proven gene construct^21^, it provides the possibility to yield large amounts of the recombinant protein by milking, and ensures safety as testified by the approved use of human recombinant proteins for the treatment of human diseases^18, 19^. Although several attempts have already been made to produce recombinant PLTP from different sources, only small amounts of PLTP could be produced, mostly for protein characterization^9–12, 28–30^, but not for PLTP administration *in vivo*. In this context, and given that in the present study one lactation period of one PLTP transgenic rabbit was able to produce as much active human PLTP as 50 liters of blood plasma from healthy subjects, milking PLTP transgenic rabbits is the first strategy so far able to provide sufficient amounts for systemic therapy. Most importantly, the resulting rhPLTP retained the main physical and functional features of native human PLTP. The administration of rhPLTP was found to diminish the harmful consequences of sepsis and dampen the uncontrolled inflammatory response. Remarkably, systemic therapy with rhPLTP only, providing a level comparable to that in normal plasma, was able to slow down bacterial growth, to reduce endotoxemia and LPS activity, to decrease circulating levels of proinflammatory cytokines and to increase survival with consistent observations when treating either PLTP-knocked out or wild-type mice. As far as mechanisms underlying the protective effect of rhPLTP against septic infection are concerned, it should be stressed that PLTP belongs to the lipid transfer/lipopolysaccharide binding protein (LT/LBP) gene family with members involved in innate immunity^2, 3^. The present study sheds new light on the key role of PLTP in the defense against infection with gram-negative bacteria with two main properties of rhPLTP that should be considered and might act in a complementary manner. First, rhPLTP was shown here, in both *Pltp*−/− and WT mice, to boost the reverse LPS transport pathway through which LPS can be disaggregated from the bacterial wall, transferred towards lipoprotein carriers, brought to the liver and finally excreted in the bile. The present observations in mice with polymicrobial infection comes in extension of earlier *in vitro* studies with plasma or cell cultures, as well as *in vivo* studies in *Pltp*−/− mice infused intravenously with purified LPS^6, 7^. Indeed, PLTP was reported to disaggregate LPS from the outer wall of gram-negative bacteria^4–8^ and to transfer LPS molecules to plasma lipoproteins for neutralization and detoxification in the bile^6, 7^. Most importantly, the present study brings the first *in vivo* evidence that PLTP can initiate the Reverse LPS transport pathway from intact, living bacteria.

Second, a new mechanism of the antimicrobial effect of PLTP emerged from the present study. Indeed, and in addition to its LPS transfer property, rhPLTP was shown for the first time to exert direct antibacterial effects, preventing the growth of Gram-negative, but not Gram-positive bacteria. This finding adds to the current knowledge about PLTP, and again, outcomes should be analyzed in the light of specific antimicrobial properties of other members of the LT/LBP gene family. For example, and as described here for the PLTP action, BPI was demonstrated earlier to exert its antimicrobial activity mainly against Gram-negative bacteria via hydrophobic interactions with LPS and membrane alterations culminating in bacterial lysis and death^31–33^. As for BPI, the results of the present study provide support for the antimicrobial effect of PLTP on Gram-negative bacteria. It is likely that this effect also relies on its affinity binding to LPS and its ability to promote changes in cell membrane morphology (see Fig. 5). Finally, by comparing *Pltp*−/− mice and wild-type mice with naturally high PLTP activity, it was further demonstrated that endogenous PLTP combines both LPS transfer properties (as demonstrated here not only in endotoxic shock, but also in a polymicrobial model of sepsis) and bacteriostatic properties (as shown *in vitro* on cultured *E*. *coli* and *in vivo* after CLP surgery).

In conclusion, the production of large amounts of rhPLTP, which remains highly active after injection into the bloodstream, opens a new strategy to prevent and treat sepsis. In this context, rhPLTP displays a number of advantages over other candidates, including the related rBPI, which has already stimulated some interest for the treatment of infection^34^. Most importantly, over the last decade most strategies to develop pharmacological interventions to treat sepsis have involved downregulation of the inflammatory process and mediators (noticeably through treatments with corticosteroids or with agents targeting LPS, Toll-like receptor 4 (TLR4), tumor necrosis factor (TNF), interleukin (IL-1) as well as platelet-activating factor (PAF)^35, 36^), whereas rhPLTP targets the very early, LPS-mediated initiating step, thus providing a unique strategy for a dual beneficial outcome: (1) the limitation of gram(-) bacterium growth and (2) the neutralization and detoxification of LPS, the culprit initiator of the inflammatory response.

## Methods

### Animals

New Zealand White (NZW) rabbits were provided by Hypharm (Sèvremoine, France). Wild-type, C57BL/6J mice were provided by Charles River Laboratories International (L’Arbresle, France). Phospholipid transfer protein (PLTP)-deficient (*Pltp*−/−) mice, generated by Dr Jiang and colleagues^37^, were on a homogenous C57BL/6J background for at least eight generations. Animals had free access to water and food. All experiments involving rabbits were performed in accordance with institutional guidelines and approved by the Poitou Charentes Ethics Committee on the Use of laboratory Animals. All experiments involving mice were performed in accordance with institutional guidelines and approved by the University of Burgundy’s Ethics Committee on the Use of Laboratory Animals. The total number of animals used was n = 268 mice (n = 74 WT mice and n = 194 *Pltp*−/− mice) and n = 26 rabbits.

### Construction of a transgenesis vector containing the sequence encoding human PLTP

#### cDNA cloning of PLTP in an intermediate vector

A retrotranslated sequence of synthetic cDNA encoding the human PLTP precursor (UniProtKB/Swiss-Prot P55058), bordered at the 5′-position by a Kozak consensus sequence and a unique *Mlu*I restriction site and, at the position 3′, by two stop codons and an *Nhe*I restriction site, was synthesized and cloned into an intermediate vector, derived from the pBluescript plasmid, containing the ampicillin resistance gene as well as the Col E1 bacterial replication origin.

#### Transgenesis Vector

The transgenesis vector used for the cloning was derived from the plasmid pPolylII, having an ampicillin resistance gene as well as the Col E1 bacterial replication origin. The vector contains an expression cassette that includes from 5′to 3′: a dimer of the 5′HS4 insulator sequence of the chicken beta-globin gene (Genbank U78775)^38^, upstream of the 6.3 kbp rabbit WAP promoter (whey acidic protein; Genbank X52564)^39^, the second intron of the rabbit beta-globin gene (Genbank V00882) containing a transcription enhancer; a second transcription enhancer (SUR 1.2.3) containing the 5′UTR sequence of the SV40 early gene fused with the R region and the start of the HTLV-1 U5 region^40^; a third transcription enhancer (Igμ2), derived from mu region of the mouse IgG heavy chain (Genbank J00440)^41^ and finally the human growth hormone transcription terminator (Genbank Ml3438). This cassette contains an *Mlu*I site and an *Nhe*I site located between the second and third transcription enhancer; it is flanked on either side by NotI sites allowing the excision of sequences of the plasmid pPolyIII.

The DNA insert encoding PLTP, recovered by *Mlu*I/*Nhe*I digestion from the intermediate vector, was inserted between the *Mlu*I and *Nhe*I sites of the expression cassette. As above, the colonies containing the recombinant vector were selected on the basis of ampicillin resistance, and then the presence of the insert was checked by analysis of the restriction fragments, and then sequencing.

### Generation of PLTP transgenic rabbits

The transgenic rabbits expressing PLTP in the mammary gland were obtained by the conventional technique of microinjection^42^.

#### Preparation of the inserts for transgenesis

The transgenesis vector containing the sequence encoding recombinant human PLTP was digested with *Not*I restriction enzyme and the insert containing the transgene was isolated on agarose gel and then purified on ElutipD (Schleicher-Schuell, Ecquevilly, France) according to the manufacturer’s instructions, ethanol precipitated and then taken up in 10 mM Tris-HCl buffer, 0.1 mM EDTA, pH 7.4.

#### Preparation of the donor and recipient rabbits

Embryo donor New Zealand rabbits, aged 20–24 weeks, were treated with subcutaneous injections of swine FSH/LH (Follicle Stimulating Hormone/Luteinizing Hormone, Stimufol®, REPROBIOL) for 3 days to stimulate follicle development. On the third day, the rabbit does were mated with male New Zealand rabbits and immediately after mating were given an intramuscular injection of Receptal® (Busereline acetate - synthetic luteinizing hormone - Intervet International B.V.). The recipient rabbits were aged 20–34 weeks. A synchronized pseudopregnancy was induced by an intramuscular injection of Receptal®.

#### Microinjection of embryos collected and implantation

On the 4th day, 18–19 h after mating, embryos were collected from the rabbit donors for the DNA microinjection procedure: DNA microinjection was carried out immediately after collecting the embryos (19–21 h after mating). The single-cell stage embryos were placed in a drop of medium under an inverted microscope equipped with Normarsky objectives and Narishige micromanipulators. Individual embryos were positioned and secured using a holding pipette. The transgene diluted to a concentration varying from l ng/µl to 6 ng/µl, in buffer containing 10 mM Tris-HCl, 0.1 mM EDTA, pH 7.4, was preferentially micro-injected into the male pronucleus of the embryo using an injection pipette.

Following the microinjection, the embryos were maintained in *in vitro* culture for 1 to 3 hours (37 °C, 5% CO_2_ in air). The quality of the micro-injected embryos was then quickly assessed under a stereomicroscope. The intact embryos were then reimplanted under general anesthesia into the lumen of the oviducts of the synchronized recipient rabbits (10–17 embryos in each oviduct), using a surgical procedure (oviducts were exteriorized by laparotomy). Parturition mostly occurred naturally 29–31 days after embryo transfer. When necessary, it was triggered by injecting Ocytovem® (Ceva) on the 31st day. The ratio of young rabbits born compared with the number of embryos reimplanted ranged from 5 to 20%.

#### Selection and characterization of the transgenic rabbits

During embryogenesis, the microinjected recombinant DNA was randomly integrated into the genome. The newborn rabbits (10 days) were tested for the presence of the transgene by a biopsy of the ear. Genomic DNA was extracted and PCR (Polymerase Chain Reaction) was performed using specific primers for the recombinant insert. Rabbits for which the transgene was detected were called “Founders F0”. The founder F0 lines were further characterized by i) analysis of the number of transgene copies integrated in their genome, and ii) determination of the number of integration sites. The number of copies of the transgene integrated into the genome of each founder F0 line was determined by quantitative PCR and Southern blotting. This number varied, depending on the line, from 1 copy per cell to a hundred copies per cell. The number of integration sites was also determined by Southern blotting. This number varied, depending on the line, from1 to 3 locations per genome.

### Milk harvesting conditions

The pups were separated from their mother overnight. To get milk the mother was retained in an apertured hammock leaving access to the mammary glands and the teats. Oxytocin (0.3 ml Ocytovem®, Ceva) was injected intramuscularly into the mother. Tubes connected to a lab-designed milking machine were placed on the teats and the pump of the machine was switched on for about 10 minutes. The milk was harvested in a specific collector. About 50–75% of the available milk was collected and the pups were then put with their mother to get the remaining milk. Duration of lactation in rabbits is 4–5 weeks. The milking took place from day 4 to day 28 when the well-being of the mother and the pup allowed it. The females were mated and milked only if their health status was in accordance with regulations on animal welfare. The milk samples were stored at −80 °C until analysis.

### Evaluation of PLTP activity in milk of the transgenic rabbits

PLTP activity in the milk of F0 transgenic rabbits was measured in milk samples using a commercially available fluorescence activity assay (Roar Biomedical Inc., New York, NY) according to the manufacturer’s instructions. This fluorimetric assay measures the transfer (unquenching) of fluorescent phospholipids from donor to acceptor synthetic liposomes. PLTP activity was expressed as the increase in fluorescence (arbitrary units (AU) of fluorescence) from the delta between 1 and 20 minutes or as the initial rate of phospholipid transfer (AU fluorescence/min).

### Purification of rhPLTP from milk of female PLTPTg_WAP_ rabbits

#### Precipitation of caseins at acid pH and room temperature

One volume of milk was mixed with 2 volumes of 0.5 mM EDTA, pH 8.0 and 7 volumes of MilliQ water. EDTA chelates calcium ions and breaks casein micelles, which may retain a portion of PLTP. The pH was gradually reduced to 4 by adding glacial acetic acid dropwise with stirring in order to completely precipitate the caseins (whose pH*i* is 4.6).

#### Clarification of the whey by low-speed centrifugation and pH neutralization

After precipitation of the caseins at acidic pH, the mixture was centrifuged for 10 min at 2000 g and 4 °C. The intermediate clarified fraction situated between the lipid supernatant and the pellet of protein was collected and neutralized with solid Tris in an amount sufficient to reach a pH of 7.4. The fraction thus obtained was filtered on a fiberglass filter (Millipore AP2004700).

#### Extraction/Purification of rhPLTP by affinity chromatography on a column of Heparin Sepharose

The clarified and filtered protein fraction was injected onto a Heparin Sepharose 6 Fast Flow column, (240 × 16 mm ID, GE Healthcare Life sciences Europe GmbH) previously equilibrated with a 20 mM Tris buffer, pH 7.4. Injection was performed at a rate of 1 ml/min with a peristaltic pump. The column was rinsed overnight with a 20 mM Tris buffer/pH 7.4. The loaded column was connected to an Äkta FPLC system. A discontinuous gradient of 20 mM Tris buffer, pH 7.4 to 20 mM Tris/1 M NaCl buffer, pH 7.4 was programmed with increments of 100 mM NaCl. Eluted fractions (A to G) were assayed for PLTP activity. The fractions showing the highest activity were pooled and filtered on a 0.22 µm filter and stored at −80 °C.

### Proteomics and mass spectrometry analysis

The previously pooled fractions containing PLTP were subjected to non-denaturing PAGE (NuPAGE 4–12% Bis-Tris gels, Novex). The protein band was visualized on staining with Coomassie Brilliant Blue G-250 (Invitrogen). The Coomassie-stained spot of interest was manually excised. The gel piece was then washed twice with 0.1 M NH_4_HCO_3_ and 100% acetonitrile (ACN) for 20 min. Reduction/alkylation was achieved by incubating the excised spot successively in 10 mM Tris(2-carboxyethyl)phosphine/0.1 M NH_4_HCO_3_ for 30 min at 37 °C and 55 mM iodoacetamide/0.1 M NH_4_HCO_3_ for 20 min. Peptide fragments were obtained after digestion with a solution of 40 mM NH_4_HCO_3_ and 10% ACN containing 5 ng/μL trypsin (Promega) for 3 h at 37 °C. The resulting peptides were acidified with 0.2% trifluoroacetic acid (TFA). Extraction from the polyacrylamide gel piece was performed by two successive incubations for 8 min in ACN. Digests were concentrated by evaporation until 6 μL. The concentrate (0.5 μL) was deposited onto a Ground Steel target (Bruker Daltonics, Bremen, Germany), mixed with 1 μL matrix solution (3.5 mg/ml α-cyano-4-hydrocycinnamic acid (CHCA) in 50/50 ACN/TFA 0.2%. Analysis was conducted using a MALDI-TOF/TOF UltrafleXtreme (Bruker Daltonics) in the automatic mode operating at 1,000 Hz in the reflectron mode. Mass calibration was done using the peptide calibration standards from Bruker Daltonics. Proteins were identified within Swiss-Prot, restricting the taxonomy to *Homo sapiens and Mammalia*. Methionine oxidation and carbamidomethyl modification of cysteines were accepted as variable and global modifications, respectively. The mass deviation tolerance was 30 ppm in mass spectrometry, and only one miscleavage was suggested.

### PLTP Activity measurements

PLTP activity was measured as previously described^43^, using a commercially available fluorescence activity assay (Roar Biomedical Inc., New York, NY) according to the manufacturer’s instructions. Briefly, samples (5 µl), fluorescent-labelled donors (3 µl) and unlabeled acceptors (50 µl), were incubated at 37 °C in a final volume of 100 µl of assay buffer in 96-well microplates. Changes in fluorescence were monitored using a Victor^3^ multilabel counter (PerkinElmer Life Sciences) with a 465 nm excitation and a 535 nm emission wavelength. This fluorimetric assay measures the transfer (unquenching) of fluorescent phospholipids from donor to acceptor synthetic liposomes. Phospholipid transfer activity was calculated from the delta of fluorescence between 1 and 20 minutes and expressed as the increase in fluorescence (arbitrary units (AU) of fluorescence) or as a percentage of WT mouse plasma activity used as a reference.

### Single LPS injection

Purified LPS of *Escherichia coli* serotype 055-B5 (Sigma Aldrich) was suspended in endotoxin-free 0.15 M sodium chloride and vigorously mixed before use. The mice received a single injection of LPS (5 or 25 mg/kg of body weight) intraperitoneally. In a separate set of experiments, blood samples were collected before (T0) and at the indicated times during the 24 h after LPS injection. Plasma was obtained by blood centrifugation (10 min, 6000 *g* at 4 °C). Samples were stored at −80 °C until analysis. In survival studies, the number of deaths was monitored twice a day for 7 days.

### Polymicrobial sepsis after cecal ligation and puncture

Cecal ligation and puncture was performed as previously described^44^. Mice were anesthetized with isoflurane and then placed on a heating pad during the surgical procedure. A midline laparotomy was made after the abdomen had been shaved and prepared with alcohol. The cecum was then exteriorized and ligated with a 4–0 suture below the ileocecal valve without causing bowel obstruction. The ligated cecum was subsequently perforated by a single through-and-through puncture with a 21-gauge needle and gently squeezed to extrude a small amount of faecal matter. The cecum was placed back into the peritoneal cavity, and the incision was closed in two layers with 6–0 sutures and wound clips. The mice were then resuscitated with 0.4 ml of saline injected subcutaneously to compensate fluid loss that occurred during the procedure. At the end of the surgical procedure the mice were returned to their cages and survival was monitored twice a day for 10 days. Separate groups of animals underwent the same procedure. Blood samples were collected before (T0) and at different times during the 24 h after CLP.

### Blood sampling

All materials were of pyrogen-free grade or made apyrogenic by overnight heating at 150 °C, and all of the reagents used were of “endotoxin-free” grade.

Blood was collected at the indicated times via retroorbital or cardiac puncture in anesthetized mice. Plasma was obtained by blood centrifugation (10 min, 6000 *g* at 4 °C), and lipoproteins were further isolated from the plasma by ultracentrifugation as previously described^6^. The *d* < 1.21 g/ml fraction constituted the Lipoprotein fraction and the *d* > 1.21 g/ml fraction constituted the Lipoprotein-free fraction. All samples were stored at −80 °C until further analysis.

### Measurements of glycaemia

Blood was drawn from the tail vein at 0, 2, 4, 6, 8 and 24 h following the CLP, and glucose concentration was determined with a glucose meter (One Touch Ultra®).

### Measurements of plasma concentrations of cytokines

Plasma concentrations of IL-6, TNFα, MCP-1, IL-1β, IFN-γ, IL-10 were quantified using a Milliplex MAP Mouse Cytokine/Chemokine Panel (# MCYTOMAG-70K, Millipore, Billerica, MA). The assays were performed according to the manufacturer’s instructions. Standards and samples were analyzed on a LuminexR® apparatus (Bio-Plex 200, BioRad, München, Germany) using the BioPlex Manager Software (Version 5, BioRad, Hercules, CA).

### LAL assay

The biological activity of LPS was quantified by the endpoint chromogenic LAL assay (QCL-1000 kit; Lonza, Walkersville, MD USA), which gives a magenta colour when positive. Briefly, 50 µl of diluted plasma (1:20), or diluted lipoprotein fraction or lipoprotein-free fraction (1:10) in endotoxin-free water were dispensed in each well of a 96-well plate. At the initial time point, 50 µl of the LAL reagent were added to each well. The plate was shaken and incubated at 37 °C for 10 minutes. Then, 100 µl of chromogenic substrate warmed to 37 °C was added to each well and incubation was extended for an additional 6 minutes at 37 °C. The reaction was stopped by adding 100 µl of a 25% solution of glacial acetic acid. Absorbance was measured at 405 nm on a spectrophotometer (Victor^3^, Perkin Elmer).

### LPS mass quantitation

LPS mass concentration was determined by direct quantitation of 3-hydroxytetradecanoic acid (or 3HM) by LC/MS/MS as previously described^26^. Briefly, samples were spiked with 5 ng of internal standard (3β-hydroxytridecanoic acid 10 mg/ml in ethanol), and then hydrolyzed with 300 μl of HCl 8 M for 4 h at 90 °C or with 75 μl of NaCl 150 mM, respectively. Free fatty acids were then extracted with 600 μl of distilled water and 5 ml of hexane. After vacuum evaporation of the hexane phase, fatty acids were dissolved in 100 μl of a 40% A/60% B eluent mixture (eluent A, ammonium acetate 5 mM pH 5.0; eluent B, acetonitrile/acetate ammonium 5 mM pH 7.3 96.7%/3.3%). Fatty acid separation was performed in an Infinity 1200 HPLC binary system (Agilent) equipped with a Poroshell 120 EC C18 100 × 4.6 mm 2.7 μm column (Agilent) set at 30 °C. The sample volume injected was 10 μl. A 7 min eluent gradient was established as follows: from 0 to 0.5 min, the flow was maintained constant at 1 ml/min of 80% B; then the proportion of B was increased linearly up to 100% in 1 min; concomitantly the flow rate was decreased to 0.5 ml/min; these conditions were maintained constant for 1 min; then the flow rate was increased to 1 ml/min for an additional 2.5 min; finally, the column was reequilibrated with 80% B at 1 ml/min for 2.5 min.

MS/MS detection was performed using a QQQ 6460 triple quadruple mass spectrometer (Agilent) equipped with a JetStream ESI source in the negative mode (gas temperature 300 °C, gas flow 10 l/min, nebulizer 20 psi, sheath gas temperature 200 °C, sheath gas flow 11 l/min, capillary 3,500 V). Nitrogen was used as the collision gas. The mass spectrometer was set up in the selected reaction monitoring (SRM) mode for the quantification of selected ions as follows: for 3-hydroxytetradecanoic acid, precursor ion 243.2 Da, product ion 59 Da, fragmentor 93 V, collision cell 9 eV; for 3-hydroxytridecanoic acid, precursor ion 229.2 Da, product ion 59 Da, fragmentor 110 V, collision cell 10 eV.

### Evaluation of bacterial burden

Blood samples were obtained from anesthetized mice via retro-orbital puncture before or 8 h after CLP and by cardiac puncture using a sterile technique 24 h after the CLP procedure. Peritoneal lavage fluids were obtained from mice 24 h after CLP by injecting sterile PBS (3 ml) through the fascia into the peritoneal cavity and gentle aspiration. For bacterial culture, peritoneal lavage fluid or blood was diluted with PBS (10-fold serial dilutions) and 30–100 µl of each dilution were spread on a Columbia agar (Oxoid) medium supplemented with 5% sheep blood. Plates were incubated at 35 ± 2 °C in anaerobic conditions for 48–72 h and then the number of colony-forming units (CFU/ml) was counted.

### *In vitro* evaluation of antimicrobial activity of purified recombinant proteins

*E*. *coli* and *S*. *aureus* growth was examined *in vitro* using a broth microdilution assay in nutrient Mueller Hinton Broth (MHB, BD 275730). *E*. *coli* (ATCC 25922) or *S*. *aureus* (ATCC 25923) were plated on Tryptic soy agar (BD236950) overnight at 37 °C. An isolated bacterial colony was used to inoculate MHB and the bacterial cultures were allowed to grow overnight at 37 °C. 100 μL of culture was used to freshly inoculate 3 ml of MHB. The suspension was then allowed to grow at 37 °C with shaking at 225 rpm for ~2 h, until a final bacterial concentration of ~10^8^ colony forming units/mL (CFU/mL) was reached (OD600 ~0.1). rhPLTP was obtained as described above and rhBPI was purchased from R&D Systems (Catalog number 7468-BP). Recombinant proteins were dialyzed in MHB and were added to a 96-well sterile microplate in order to get two-fold serial dilutions in MHB. Next, 50 μl of inoculum containing 10^6^ CFU/ml of *E coli* or *S*. *aureus* was added to each well, so that each well contained 50 µl of rhPLTP or rhBPI solution and 50 µl of cell suspension. Then, the microplates were incubated at 37 °C. Wells containing MHB with and without bacteria were used as growth and sterility controls, respectively. Bacterial growth was determined at 6 h after incubation by measuring absorbance at 600 nm (OD600).

### Transmission electron microscopy (TEM)

Cells were pelleted by centrifugation for 5 min, at 3000 g and 4 °C. After washing with Sorensen’s phosphate buffer (0.1 M, pH 7.4), the pellets were re-suspended with a solution of 4% [v/v] paraformaldehyde and 1.5% [v/v] glutaraldehyde in Sorensen’s phosphate buffer. After fixation for 45 min at room temperature, samples were washed four times with Sorensen’s phosphate buffer at 4 °C and embedded in low-melting-point agarose (1.5–2%). The agarose pellet was solidified at 4 °C and cut into small cubes of about 1 mm edge length. Samples were post-fixed with 1% osmium tetroxide in Sorensen’s phosphate buffer in the dark for 1 h at room temperature. Samples were dehydrated with graded ethanol and propylene oxide and embedded in EMBed-812 resin. Ultrathin sections were cut with an ultramicrotome (Reichert Ultracut E, Leica, Rueil-Malmaison, France) and transferred to copper/palladium grids. The grids were contrasted with uranyl acetate and lead citrate and observed on a Hitachi H7500 TEM (Hitachi Scientific Instruments Co., Tokyo, Japan) operating at 80 kV and equipped with an AMT camera driven by AMT software (AMT, Danvers, USA).

### Histological Examination

Liver, and kidney segments were fixed in 10% (v/v) phosphate-buffered formalin (pH 7.4) for 48 h and then embedded in paraffin. Five-µm-thick sections were stained with hematoxylin and eosin (HE) and viewed with a digital slide scanner (NanoZoomer, Hamamatsu, Japan) at x400 magnification. All tissue sections were evaluated blindly by a pathologist.

### Statistics

Data are presented as the mean ± sem. All data were analyzed using GraphPad Prism 7 software (GraphPad Software Inc.). The differences in survival rates were analyzed by Kaplan-Meier plot and the statistical significance was determined using log-rank test or *χ* ^*2*^ test as indicated. For pairwise comparison of experimental groups, unpaired *t* test or Mann-Whitney test was performed. Differences between multiple groups were analyzed by one way ANOVA with Tukey’s multiple comparisons test for normally distributed data or by the Kruskal-Wallis non-parametric test followed by Dunn’s multiple comparison test. For all statistical analyses, a P value of 0.05 was considered significant.

### Data availability

The authors declare that the data supporting the findings of this study are available from the corresponding author upon request.
